# Supplementary material for: Expression of interleukin-4 in schistosomiasis is influenced by age
Source: PLoS Negl Trop Dis. 2025 Jun 17;19(6):e0013176. doi: 10.1371/journal.pntd.0013176 (PMC12173192; doi:10.1371/journal.pntd.0013176)
Supplement: Attachment — Submitted filename: Review comments_250103.docx [file pntd.0013176.s003.docx]

The study entitled “Immunological biomarkers and correlates of schistosomiasis in Mulobezi district: a cross-sectional study” addresses an important aspect of public health issue in Zambia, providing insightful data on schistosomiasis integrating immunological biomarkers, risk factors and demographic data. The manuscript is structured well, however there are several drawbacks in this paper which are addressed below.

1. The introduction effectively frames schistosomiasis as a significant public health issue in Sub-Saharan Africa, linking it to the disease burden in Zambia. However, the manuscript lacks the broader aspect of how findings from this region can contribute to broader global health strategies. It also lacks adequate explanations (and citations) why IL-4 and IL-10 were chosen as key biomarkers or their known roles in schistosomiasis. There is no explanation on how studying those cytokines can advance diagnostics, treatment/prevention strategies. The introduction section needs to be rewritten.

2. The major drawback for this paper is the low sample size of 143, which may limit the statistical power and generalizability of the findings reported, as also reported as one of the limitations by the authors. Is there any future plan to include more diverse and bigger sample size?

3. The method of ELISA lacks specific details like manufacturer information, sensitivity and assay protocol.

4. Authors have stated an increase in IL-4 level in patients with Schistosomiasis, but IL-10 level did not change. Lack of IL-10’s association with schistosomiasis is reported but not discussed in detail. Authors could provide a brief rationale in relevance to schistosomiasis or similar parasitic diseases.

5. “Schistosomiasis diagnosis was confirmed through urine microscopy using filtration methods”: this sentence does not sound correct. Please rewrite.

6. Line 61: the last keyword (*Schistosoma haematobium*) is missing.

7. Genus and species name should be indicated in italics throughout the manuscript.

8. There are too many grammatical mistakes to point out. It would be wise to edit the paper by a native English speaker.
