# Supplementary material for: Expression of interleukin-4 in schistosomiasis is influenced by age
Source: PLoS Negl Trop Dis. 2025 Jun 17;19(6):e0013176. doi: 10.1371/journal.pntd.0013176 (PMC12173192; doi:10.1371/journal.pntd.0013176)
Supplement: Attachment — Submitted filename: Rebuttal Letter (1).docx [file pntd.0013176.s004.docx]

Date: 25^th^ April 2025,

To: EditorPLOS Neglected Tropical Diseases

**Subject: Response to Reviewer Comments for Manuscript ID PNTD-D-24-01888**

We sincerely thank you for the time and effort devoted to reviewing our manuscript. We appreciate the constructive comments and suggestions, which have greatly helped us improve the clarity and scientific rigor of our work.

We have addressed each comment carefully and revised the manuscript accordingly. To achieve this we redesigned the study, and we have rewritten a large section of the manuscript.

Below, we provide a point-by-point response to the reviewers’ comments. We hope the revised version meets your expectations and is now suitable for publication in PLOS ONE.

**REVIEWERS COMMENTS**

1. **REVIEWER # 1 COMMENTS**

**General Comment:** *"The study entitled 'Immunological biomarkers and correlates of schistosomiasis in Mulobezi district: a cross-sectional study' addresses an important public health issue in Zambia, providing insightful data on schistosomiasis by integrating immunological biomarkers, risk factors, and demographic data. The manuscript is structured well, however, there are several drawbacks which are addressed below."*

***Comment 1.1:*** *"The introduction effectively frames schistosomiasis as a significant public health issue in Sub-Saharan Africa, linking it to the disease burden in Zambia. However, the manuscript lacks the broader aspect of how findings from this region can contribute to broader global health strategies. It also lacks adequate explanations (and citations) as to why IL-4 and IL-10 were chosen as key biomarkers or their known roles in schistosomiasis. There is no explanation on how studying these cytokines can advance diagnostics, treatment, or prevention strategies. The introduction section needs to be rewritten."*

**Response:** We appreciate this valuable suggestion. We have rewritten the Introduction section to provide a broader global health context and further justification for the selection of IL-4 and IL-10, including additional citations explaining their role in schistosomiasis.

***Comment 1.2****: "The major drawback for this paper is the low sample size of 143, which may limit the statistical power and generalizability of the findings reported, as also reported as one of the limitations by the authors. Is there any future plan to include more diverse and bigger sample sizes?"*

**Response:** We acknowledge the limitation of our sample size and have explicitly discussed future plans for expanding our study with a larger and more diverse sample).

***Comment 1.3:*** *"The method of ELISA lacks specific details like manufacturer information, sensitivity, and assay protocol."*

**Response**: We have now expanded the Methods section (Lines 101–-108) to include manufacturer details, sensitivity levels, and a summary of assay protocol.

**Comment 1.4:** *"Authors have stated an increase in IL-4 level in patients with schistosomiasis, but IL-10 levels did not change. Lack of IL-10’s association with schistosomiasis is reported but not discussed in detail. Authors could provide a brief rationale in relevance to schistosomiasis or similar parasitic diseases."*

**Response:** We appreciate the reviewer's insightful comment regarding the lack of association between IL-10 levels and schistosomiasis in our study. We have updated our discussion on page 22.

***Comment 1.5:*** *"Schistosomiasis diagnosis was confirmed through urine microscopy using filtration methods": this sentence does not sound correct. Please rewrite."*

**Response:** The sentence has been revised in the Methods section (Page 2, Line 34-35) for clarity.

***Comment 1.6:*** *"Line 61: the last keyword (Schistosoma haematobium) is missing."*

**Response:** We have added the missing keyword in the Abstract (Page 3, Line 60).

***Comment 1.7****:* *"Genus and species names should be indicated in italics throughout the manuscript."*

**Response:** We have ensured that all occurrences of Schistosoma haematobium and related genus and species names are italicized throughout the manuscript.

***Comment 1.8****: "There are too many grammatical mistakes to point out. It would be wise to edit the paper by a native English speaker."*

**Response:** We have thoroughly proofread and revised the manuscript to correct grammatical and typographical errors.

**COMMENTS ON METHODS**

2. **REVIEWER # 2**

***Comment 2.1****: "The inclusion criteria for study participants were not rigorous enough. For example, previous infections are not considered, and previous infections may be important factors that affect immune markers."*

**Response:** Thank you for your insightful comment regarding the inclusion criteria and the potential influence of previous infections on immune markers. This will be highlighted as a limitation and its inclusion will be recommended for a later study.

***Comment 2.2: "The details of ELISA were not provided."***

**Response**: We have now expanded the Methods section (Lines 102–108) to include manufacturer details, sensitivity levels, and a step-by-step assay protocol.

**Comment 2.3*:*** *"Why did you only choose IL-4 and IL-10?"*

**Response:** IL-4 and IL-10 were chosen due to their significant roles in schistosomiasis immunopathology. IL-4 is critical in Th2 response and eosinophil recruitment, while IL-10 regulates inflammation. We have rewritten the Introduction section (Lines 61-69) to provide a broader global health context and further justification for the selection of IL-4 and IL-10, including additional citations explaining their role in schistosomiasis.

**Comment 2.4*:*** *"Did you think the factors you investigated were enough?"*

**Response:** We acknowledge that additional immunological and environmental factors could enhance the study. We have added a discussion on potential additional variables in future studies (Discussion, Lines 359–363).

**Comment 2.5*:*** *"Relative to the high prevalence of schistosomiasis, the inclusion of study participants is insufficient, and the results are less convincing."*

**Response:** Thank you for your comment regarding the sample size and its implications for the study’s conclusions. We acknowledge that a larger sample size could enhance the robustness and generalizability of our findings, particularly given the high prevalence of schistosomiasis observed. To mitigate thisb we have redesigned the study, put less variables to get the best results.

3. **REVIEWER # 5**

**Comment 4.1:** *"In the methods section of the abstract (and line 168), please specify whether your tests were one (> or <) or two-sided."*

**Response**: We have now specified in the Methods section of the Abstract and Lines 36 and 130 that the tests conducted were two-sided statistical tests.

**Comment 5.2:** *"In the methods section of the abstract, please consider replacing ‘A logistic regression model (bivariate and multivariable) was used to …’ with ‘Unadjusted and adjusted logistic regression models were used to …’ or ‘Simple and multiple logistic regression models were used to …’. In lines 167-168, you write, ‘Univariable and multivariable logistic regression was used to examine the factors …’ This is fine, but you need to be consistent in your usage of these terms (correct lines 219-221 in the results section because you alternate between -ate and -able suffixes and they mean different things)."*

**Response**: We appreciate this suggestion. We have made the recommended changes for consistency in terminology throughout the Methods section of the Abstract and Results section to maintain uniformity in the use of unadjusted and adjusted logistic regression models.

**Comment 5.3*: "Did you run a sample size calculation based on accurate assumptions? Can you provide a power analysis? It seems like you included a lot of variables in your multivariable model given the small sample size."***

**Response:** Thank you so much for the insightful comment. We have redesigned the study to include less variables

4. **REVIEWER # 6**

**Comment 5.1*:*** *"Is the study design appropriate to address the stated objectives?" Yes, but I expected the authors to use a greater number of immunological markers and a larger sample size categorized by population type.*

**Response:** Thank you for your valuable feedback regarding the study design and the scope of immunological markers. We acknowledge that including a greater number of immunological markers and a larger, stratified sample size would provide deeper insights into the immunological correlates of schistosomiasis. However, given resource constraints and the study’s cross-sectional design, we focused on key cytokines (IL-4 and IL-10) and hematological markers known to play significant roles in schistosomiasis-related immune responses. Additionally, while our sample size was limited, we ensured a diverse participant selection from different communities to enhance representativeness.

We have acknowledged this limitation in the Discussion section, emphasizing the need for future studies with expanded immunological profiling and a larger, stratified sample to explore immune responses across different population subgroups. We appreciate your insightful suggestion and believe these clarifications enhance the manuscript.

**Comment 5.2: *"****Is the population clearly described and appropriate for the hypothesis being tested?" Yes, it is clearly described, but it is not entirely appropriate.*

**Response:** Thank you for your feedback. While our study population was clearly described and appropriately selected to explore schistosomiasis-related immunological markers, we acknowledge that a more diverse or stratified sample could have further strengthened our findings. We have now addressed this in the Discussion section, emphasizing the need for future studies to expand recruitment across different population groups to enhance generalizability.

**Comment 5.3*:*** *"Is the sample size sufficient to ensure adequate power to address the hypothesis being tested?"*

**Response:** Thank you so much. We have redesigned the study and reduced the variables included in the model to make it more robust and reliable.

**Comment 5.4:** *"Were correct statistical analyses used to support conclusions?"*

**Response:** Thank you for the question. Yes, appropriate and standard statistical analyses were employed to support the study’s conclusions. All hypothesis testing was conducted using two-sided tests, and the choice of statistical methods was guided by the nature of the data and the research questions to ensure valid and reliable inferences.

**Comment 5.5: "***Please provide bibliographical support for the following:*

*Why did you choose that sample size, and how would this limit the conclusions of your study? Susceptibility to infection according to the sex and age of the patients."*

**Response:** Thank you so much for the comment. The sample size was determined based on feasibility and the available population during the study period, which is a common approach in observational studies with limited resources. While this may limit the generalizability of the findings, the sample still allowed for meaningful subgroup analyses.

***Comment 5.5:*** *"I suggest adding a figure showing the study area."*

**Response:** Thank you for your thoughtful suggestion. Unfortunately, we are not able to put one currently. We will endeavor to so in future studies.

**Comment 5.6***:* **"***I suggest indicating how many blood and urine samples were taken per person. Indicate how the samples were taken, and how the analysis of the samples was performed. Also, mention which were the positive and negative controls in the analysis of the samples."*

**Response:** Thank you so much. We appreciate the suggestion. Each participant provided 2 blood and one urine sample, collected using standard venipuncture and midstream clean-catch techniques, respectively. Samples were processed according to standard protocols which we have highlighted in the methodology section. Commercially available positive and negative controls were included in all ELISA runs and other assays to ensure accuracy, reliability, and quality control throughout the analysis process.

**RESULTS.**

**Reviewer #2**

**Comment*:*** *Although the analysis presented matches the analysis plan, it is not appropriate due to the shortcomings of the design. Moreover, no novel findings were produced from this study.*

**Response:** Thank you so much, we have since redesigned our study and made it more robust.

**Reviewer #5.**

**Comment 1:** *Consider including the p-values or 95% CIs throughout the Results section of the abstract. Including both is redundant***.**

**Response:** We have revised the Results section of the abstract to ensure clarity and consistency, reporting only the 95% CIs as appropriate

**Comment 2***: Replace “likelihood” with “higher odds” when referring to odds ratios*.

**Response**: We have corrected the terminology throughout the manuscript to reflect “higher odds” instead of “likelihood” when referring to odds ratios.

**Comment 3***: In the Synopsis, replace “significant” with “substantial” to avoid confusion with statistical significance.*

**Response:** We have replaced “significant” with “substantial” in the Synopsis section

**Comment 4*:*** *Line 120: Replace “march” with “March” and insert the last day for June 2024.*

**Response:** Thank you. The correction was made as per recommendation.

**Comment 5***: Did you control/adjust for community in your multivariable logistic regression model?*

**Response:** Thank you for your insightful comment. In our multivariable logistic regression model, we did not include the community as a covariate. However, we recognize that differences in environmental exposures, sanitation infrastructure, and water sources between communities could influence schistosomiasis risk and immune responses.

To address this, we have acknowledged this limitation in the Discussion section, noting that future studies should consider adjusting for community-level factors to better account for potential variations in exposure and disease prevalence. Despite this, our model included key individual-level risk factors, such as water source and protective behaviors, which partially capture differences between communities.

**Comment 6*:*** *Did you consider any two-way interactions/effect modifications?*

**Response:** Thank you for your insightful question. In our analysis, we did not include interaction terms or assess effect modification explicitly. However, we acknowledge that interactions between key variables such as sex, age, and immune markers could influence the relationship between schistosomiasis infection and immunological responses.

**Comment 7:** *Define symptoms related to schistosomiasis. Would there be any benefit to examining them separately?*

**Response:** Thank you for your comment. Since we redesigned our study, this variable was removed from the study.

**Comment 8*:*** *Ensure consistency in date formatting throughout the manuscript*.

**Response:** We have standardized the date formatting throughout the manuscript.

**Comment 9:** *In the results section, be consistent in if/how you present the IQR text—as (Q1, Q3) or the difference Q3-Q1—personal preference for the former.*

**Response:** We have standardized the presentation of IQR as (Q1, Q3).

**Comment 10*:*** *Also, be consistent in how your present summary stats for the categorical variables—personal preference for frequency (percent).*

**Response:** Thank you for the comment, we have standardized the summary stats frequency (percent) as the preference.

**Comment 11*.*** *In Tables 1 and 2 of the results section, you report only n=1 employed participant. It seems odd to identify this as an occupation variable. n=94 are in school, so there seems to be overlap with the education variable. Consider combining these two variables, given your small sample size, in your models. Consider combining secondary and tertiary education in your models, too. Also, consider combining rarely and never contact with contaminated water in your models. Also, replace “domestic us” with “domestic use” in table 1.*

**Response**: Thank you. Since we have redesigned our study, these variables are no longer part of the study.

**Comment 12*.*** *In Table 1, what constitutes “protective measures” here?*

**Response:** We acknowledge the need for clarity on this term. However, we have since removed this variable from our study.

**Comment 13***: What were the other medical conditions that n=15 participants were diagnosed with?*

**Response:** Thank you, but we have since removed this variable from our study.

**Comment 14**: In Table 2, you have some cells with 0-2 participants. Consider collapsing categories for these variables before running statistical tests and fitting models.

**Response:** Thank you, however, we have since removed this variable from our study.

**Comment 15:** In Table 2, the p-value for “treated with schistosomiasis” is incorrect given the cell counts. Please double check all your results. It should be <0.0001.

**Response:** We appreciate the reviewer’s attention to detail.

Thank you, but we have since removed this variable from our study.

**Comment 16**: In your multivariable model (table 3), you use a lot of variables given the small sample size. Please run a power analysis.

**Response:** Thank you, we have since redesigned the study and included less variables.

**Reviewer number 6**

**Comment 1:** Does the analysis presented match the analysis plan?
The analysis presented does not fully match the analysis plan. In the methods, they mention the use of Chi-square, but in the results, there is no value for it. Therefore, I suggest indicating the Chi-square values. Also, I suggest indicating the results of normality tests used. Please.

**Response: Thank you so much. We have since redesigned the study and the current analysis plan is as planned. We have also mentioned the normality test used (Shapiro-Wilk test).**

**Comment 2**: Are the results clearly and completely presented? I suggest representing the relevant results in the tables using plots. Also, I recommend removing Figure 1 if it is not a result of the original research or conceptual interpretation of the discussion process carried out in the paper. In addition, it is necessary to add the reference(s) that allowed this figure to be constructed.

**RESPONSE: We have revised accordingly. Thank you for the suggestion.**

**Comment 3:** Are the figures (Tables, Images) of sufficient quality for clarity? Yes, but keep in mind representing the relevant results on plots. Please.

**Response: We have revised the results and edited accordingly.**

**COMMENTS ON CONCLUSION**

**Reviewer #2**: although you discussed the limitations of your study, it needs significant improvements in design and analysis.

**Response:** Thank you so much for the insightful comment. We have since redesigned the study to strengthen it.

**Reviewer #6**: Are the conclusions supported by the data presented?

Please. Limit the conclusion according to the scope and limitations of your research.

-Are the limitations of analysis clearly described?

Yes, in the discussion, but no in the conclusions.

-Can the authors discuss how these data can help advance our understanding of the topic under study?

**Response**: The conclusion has been updated according to the recommendations.

**Summary and General Comments**

**Reviewer #1**: This manuscript reported the association between potential risk factors and schistosomiasis in Mulobezi District based on a cross-sectional study. The topic falls into the scope of the journal. The research is well presented. However, I have several questions before it can be published.

1. **Title: ‘**correlates’ looks weird. Could be ‘risk factors?

**Response:**

2. **Abstract:** Line 37- 38. “A logistic regression model (bivariate and multivariable) was used to…”, which is inconsistent with the method (Univariable and multivariable logistic regression) described in the method section and result section

**Response:** We appreciate this suggestion. We have made the recommended changes for consistency in terminology throughout the Methods section of the Abstract, Line 37, and Results section (Lines 231-232) to maintain uniformity in the use of unadjusted and adjusted logistic regression models.

3. **Introduction:** Please briefly provide some background on biomarkers for Schistosomiasis

**Response:** We appreciate this valuable suggestion. We have rewritten the Introduction section (Page 5, Lines 95–115) to provide a broader global health context and background biomarkers for schistosomiasis.

4. **Methods: can the authors obtain some other environmental and biological data, such as land cover, meteorological data, snail population, water exposure data?**

**Response:** Thank you for your valuable suggestion. While our study primarily focused on immunological biomarkers and risk factors associated with schistosomiasis, we acknowledge the importance of incorporating environmental and biological data, such as land cover, meteorological data, snail population, and water exposure data. Unfortunately, these data were not collected in our current study. However, we recognize their significance in understanding disease transmission dynamics and plan to incorporate such data in future studies to provide a more comprehensive analysis of schistosomiasis risk factors.

5. **In the regression model, how did the authors select the final model? Is the multicollinearity issue considered?**

**Response:** The final model was selected using a stepwise approach, where variables with biological plausibility and statistical significance in the univariable analysis (p < 0.05) were considered for inclusion in the multivariable logistic regression model. However, gender was included in the final model despite not being statistically significant in the univariable analysis to account for potential confounding effects. We acknowledge that we did not explicitly assess multicollinearity using statistical methods such as the Variance Inflation Factor (VIF). In future studies, we will incorporate multicollinearity diagnostics to enhance the robustness of our regression modeling.

6. **Results: what are the associations for different age groups (children, elderly). It is likely that young children and elderly population are vulnerable to this infection.**

**Response:** Thank you for your insightful comment. We have since redesigned the study to compare adults (≥15 years) and children (<15 years), we believe this adds more insight.

7. **What are confounders considered in the multivariable logistic regression?**

**Response:**

In our multivariable logistic regression analysis, we considered potential confounders based on biological plausibility and prior literature on schistosomiasis risk factors. The key confounders included age and hemoglobin levels. Gender was included in the final model despite not being statistically significant in univariable analysis to account for its potential confounding effects.

While we aimed to control for relevant confounders, we acknowledge that other unmeasured factors, such as socioeconomic status, nutritional status, and co-infections, could also influence the associations observed. Future studies with more comprehensive data collection will help refine the analysis and further minimize residual confounding**.**

8. **Discussion: The authors found the significant association between using river water likelihood of schistosomiasis infection. Can the authors elaborate what the pathways to expose the parasite through river water, such as drinking, recreational activities, or clothe and hand washing? Is the water boiled or treated before drinking?**
9. **Response:** Thank you for your insightful comment. We have since redesigned our study and this variable has been removed.**Can the authors discuss the potential influence on the results by confounders?**

**Response:** Thank you for your insightful comment. We acknowledge that potential confounders could have influenced the associations observed in our study. We have updated the discussion on confounders (lines 300-304)

10. **Some associations are staggering, for example: “the presence of symptoms related to schistosomiasis greatly increased the likelihood of infection (AOR = 108.07, 95% CI: 11.24-1037.61, p < 0.001)”. Can the author explain what the possible reasons are?**

**Response**: Thank you for highlighting this point. We have since redesigned our study and removed this variable from our study.

**Reviewer #4**

**Comment 1:** Line 96; the sentence is incomplete and uncomprehensive.

**Response:** Thank you for your comment. . We have since revised the manuscript and removed the sentence.

**Comment 2:** Line 154; Please provide the code book reference and some details.

**Response:** Thank you for the comment. The code book reference and details have been provided.

**Comment 3**: Line 194; The eosinophil count representation is confusing, either correct or please provide detail.

**Response**: We acknowledge the potential confusion in how the eosinophil count was presented. We have clarified the unit of measurement and ensured consistency throughout the manuscript. The eosinophil count is reported in x10³/µL, and we have ensured that the values presented in tables and text are correctly formatted to avoid misinterpretation.

**Comment 4:** In the discussion, line 242-243, the author's state about the prevalence of disease is more in females in comparison to males, and later provide the describe the reasoning about it. The sample size is too small for the statement and probably the statement could be described with the reasoning together at the same place.

**Response**

We recognize that the sample size (n = 143) may be too small to make definitive conclusions about gender differences in schistosomiasis prevalence. **We have since removed prevalence from our manuscript.**

Conclusion

We extend our sincere gratitude to the reviewers and editors for their invaluable feedback, which has significantly strengthened the scientific rigor and clarity of our manuscript. Key revisions include expanding the Introduction to contextualize our findings within global health strategies, with added justification for selecting IL-4 and IL-10 as biomarkers, supported by citations detailing their roles in schistosomiasis immunopathology. The Methods section was revised to include ELISA protocol specifics (manufacturer details, sensitivity, controls) and clarify diagnostic procedures. To address concerns about statistical power, we redesigned the study to prioritize fewer variables, enhancing model robustness, and explicitly discussed plans for future larger-scale studies. We updated the Discussion to elaborate on the lack of IL-10 association and revised terminology for consistency (e.g., "higher odds" vs. "likelihood"). Grammatical errors were rectified, and species names were italicized throughout. Statistical analyses were refined, including standardized reporting of confidence intervals, IQR formatting, and acknowledgment of design limitations. Finally, we restructured tables, removed non-essential variables, and strengthened the conclusion to align with the study’s scope. These revisions collectively enhance the manuscript’s contribution to understanding schistosomiasis correlates and inform future research directions.
